# Preference for Contraceptive Implant Among Women 18–44 years old

**DOI:** 10.1089/whr.2021.0113

**Published:** 2021-12-15

**Authors:** Joana E. Matos, Bridget L. Balkaran, Jillian Rooney, Simone Crespi

**Affiliations:** ^1^ Cerner Enviza, New York, New York, USA.; ^2^ Organon, Jersey City, New Jersey, USA.

**Keywords:** long-acting reversible contraceptives, implant, unintended pregnancies

## Abstract

***Introduction:*** This study aimed to understand women's preferences regarding the subdermal contraceptive implant and to assess the proportion of women who would be underserved (with increased unintended pregnancies as the consequence) by not providing implant access equal to that of uterine-based long-acting reversible contraceptive methods (LARCs).

***Methods:*** A total of 1,200 women aged 18–44 years old (mean: 30.42 ± 7.67 years) participated in a U.S. cross-sectional online survey. To qualify for the study, women had to be sexually active with a male and not pregnant or trying to get pregnant at the time of the study. Women who had undergone a hysterectomy, a bilateral salpingo-oophorectomy, or a tubal ligation, and women with general infertility or those with a vasectomized partner were excluded. Descriptive analyses were conducted and weighted estimates, projecting to the total U.S. population were also provided.

***Results:*** The majority of women (72.6%) reported that they would be willing to switch to a LARC, should it be readily available to them. Considering those women who already use an implant and those who would be willing to switch to it, 58% of women would be underserved by not being provided equal access to the subdermal implant. This reduced availability of this type of LARC may alone elevate the number of unintended pregnancies in the United States by ∼8% of all pregnancies per year.

***Conclusion:*** Thus, making all the available contraceptive methods and maintaining access to LARCs would help reduce unintended pregnancies and better serve women and their family planning needs.

## Introduction

Modern contraception gives women and opposite-sex couples the tools to control when or whether they would like to have children. Still, in the United States alone, there are nearly 3 million unintended pregnancies per year, accounting for ∼45% of all pregnancies.^1^ Importantly, from those 3 million of unintended pregnancies, about 45% are results of contraceptive failure such as condom break.^2^ The rate of unintended pregnancy has decreased substantially though, over the years, most likely due to an increase in the use of highly effective long-acting reversible contraceptive methods (LARCs), as well as all contraceptive methods in general.^1,3^

LARCs, which include intrauterine devices (IUD) and systems (IUS), and subdermal implants, are highly effective and are associated with high rates of method continuation.^4^ However, the most commonly used contraceptive method in the United States is the oral contraceptive pill.^5^ Daily compliance is required to achieve optimal efficacy for the prevention of pregnancy. However, real-world failure rates are relatively high (estimated 7% vs. 0.3% of optimal efficacy if use was consistent and correct).^2^ Because LARCs are not user dependent, typical-use pregnancy rates for LARCs are much lower (0.8% with copper IUD, 0.2% with levonorgestrel-20 IUD and 0.1% with implant) when compared with oral contraceptives (7%).^4^

Even though the use of contraceptives remained steady (88%) between 2014 and 2016 among sexually active females, the use of short-acting reversible contraceptive methods among users decreased from 32% to 28%, whereas the use of LARCs rose from 14.3% to 17.8%. Contraceptive implant use increased from 2.6% to 4.3%.^6^ The most notable increase in implant use has been among women aged 15–19 years, where implant use rose from 6% to 16%.^6^

In general, women who find an IUD or implant highly acceptable and who know someone who has used the method are more likely to choose those respective methods at the end of their healthcare provider visit.^7^ Access to IUDs is relatively easy, as the majority of obstetricians and gynecologists (ob/gyns) reported providing IUDs in their practice (91%), according to a survey of providers conducted in 2017. In contrast, the same survey showed that the accessibility to contraceptive implants is lower, as only 71% ob/gyns reported offering this option.^8^

Professional Guidelines issued by the American College of Obstetricians and Gynecologists (ACOG) as well as the American Academy of Pediatrics (AAP) support the use of LARCs and recommend that IUDs and implants be offered routinely as safe and effective contraceptive options for most women.^9,10^ In 2016, the U.S. Department of Health and Human Services (HHS) issued guidelines for Contraceptive Care Measures, including the National Quality Forum (NQF) #2904, which was designed with the objective of identifying areas where women might have limited or no access at all to LARCs, in an attempt to minimize barriers to access.

Low rates of LARC use might indicate that there are barriers to LARC provision, which could include women's or clinicians' lack of knowledge, financial constraints or logistic issues.^11^ In fact, programs aimed at reducing such barriers, such as the Contraceptive CHOICE Project and the Colorado Family Planning Initiative, have shown that increasing access to LARCs leads to a significant increase in their use, which in turn has a powerful effect on reducing unintended pregnancy.^12,13^

In one estimate, rates of unintended pregnancy in the United States in 2014 would have been 68% higher without publicly funded family planning services.^14^ Another study estimated that publicly supported contraceptive services reached 8.9 million low-income women in 2010, which contributed to an estimated 2.2 million fewer unintended pregnancies and a net public saving of $10.5 billion.^15^

Furthermore, defunding family planning services, such as Planned Parenthood, has been shown to have deleterious effects on LARC usage and lead to an increase in unintended pregnancies.^16^ The exclusion of Planned Parenthood affiliates from Texas' fee-for-service family planning program in 2013 was associated with a 35.5% reduction in the number of pharmacy and medical claims for LARCs in addition to an increased rate of childbirth.^16^ For payer systems to maintain optimal access and achievement of the quality goals, access to women's preferred LARCs should be maintained.

In the face of potential changes in the policy landscape in the United States, and a renewed focus on women's choices, priorities, needs, and preferences understanding women's stated preference for contraceptive methods is important. The main goal of this study was to contribute to an understanding of women's preferences regarding the subdermal contraceptive implant and to estimate the proportion of women who would be underserved (with increased unintended pregnancies as the consequence) by not providing implant access equal to that of uterine-based LARCs and other contraceptive options.

## Methods

### Cross-sectional survey

Women aged 18–44 years old completed an online cross-sectional survey (Supplementary Data), between June and August 2019 in the United States. The respondents of this study were sourced from the U.S. National Health Wellness Survey (NHWS) database, which is maintained by Kantar Profiles, and from the general population through an ailment panel also maintained by Kantar Profiles. Respondents were recruited through opt-in e-mails, co-registration with panel partners, e-newsletter campaigns, and online banner placements.

To qualify for the study, women had to be sexually active with a male and not pregnant or trying to get pregnant at the time of the study, regardless of their current method of contraception. Women who had undergone a hysterectomy, a bilateral salpingo-oophorectomy (surgical removal of both ovaries), or a tubal ligation, as well as women with general infertility, or those with a vasectomized partner were excluded from the study. All respondents provided informed consent electronically and were compensated for their time through reward points.

The study was reviewed and received exemption status by the Pearl IRB (Indianapolis, IN; 19-KANT-191). Survey collected data on sociodemographic characteristics (age, race, ethnicity, marital status, education, employment status, household income, insurance status, and body mass index [BMI]), current and past contraceptive methods (multiselect—birth control arm implant, IUD/IUS, birth control shot, birth control vaginal ring, birth control patch, birth control pill, male condom, female condom, diaphragm, cervical cap, spermicide, fertility awareness methods [rhythm, calendar methods], withdrawal [pull-out method], breastfeeding as birth control, outercourse and abstinence, none).

Definitions for each type of contraceptive method were provided in the survey and were based on the definitions published by the Planned Parenthood Federation of America.^17^ Women were also asked about their willingness to switch contraceptive methods, and willingness to switch to an IUD/IUS and to the birth control implant. Preferences of contraceptive methods should the implant not be available were assessed.

### Statistical analysis

Descriptive statistics were conducted on all study variables. Means and standard deviations were recorded for continuous and count variables. Categorical variables and prevalence estimates were described in the form of counts and proportions. Subgroup analysis stratifying the sample by age groups was conducted descriptively. In addition to raw estimates, weighted estimates, projecting to the total U.S. population are also provided.

The NHWS database uses a quota sampling framework to match the demographic distribution of the adult population in the United States based on governmental statistics. For this project, sampling weights (based on age, contraceptive use, and race/ethnicity), derived from the National Survey of Family Growth (NSFG) 2015–2017 data, were used to calculate the weighted prevalence of the various contraceptive methods for the corresponding population of women aged 18–44 years.

Prevalence of being willing/open to switching to another form of contraceptive method and willingness to switch to the birth control implant or to an IUD/IUS were provided, as raw and weighted estimates. The proportion of women who prefer a nonuterine versus uterine option were assessed by determining the proportion of women who are not at all willing to switch to an IUD/IUS. Reasons not to use an IUD/IUS were reported with frequency and percentage of respondents selecting each option.

Women who already use the implant and women who were potential implant users (*i.e.*, willing to switch to an implant) were asked about their second choice of contraception should the implant not be available. The prevalence of the choices was determined for each contraceptive method and were provided in counts and proportions. In addition to raw estimates, weighted estimates of the prevalence of the various second choice contraceptive methods, projecting to the total U.S. population are also provided, utilizing the NSFG sample weights to extrapolate beyond the recontact sample.

### Proportion of underserved U.S. women

The potential proportion of women in the United States who could be underserved by not being provided equal access to the subdermal implant contraceptive was estimated by combining the proportion of women who currently use a subdermal implant and the proportion of women with some degree of willingness to switch to an implant. Weighted estimates are provided.

### Prevalence of potential unplanned pregnancies

Failure rates associated with different types/classes of contraception have been published in Trussell and Aiken.^2^ For some of the contraceptive methods, there are significant differences between failure rates during “as commonly used” and failure rates during “consistent and correct” use. Typically, methods that are more user dependent will bear a larger difference between “as commonly used” effectiveness and “consistent and correct use” effectiveness.^2^

For this analysis, “as commonly used” published failure rates, which are defined as the “percentage of women experiencing an unintended pregnancy during the first year of typical use” were used. Respondents who report using “Abstinence and Outercourse” as their current method of contraception were described using frequencies and percentages. Because they are responding with abstinence and outercourse as their exclusive form of contraception and thus, minimal chance of conceiving, these respondents were dropped from further analyses describing unintended pregnancies.

To estimate the number of potential unintended pregnancies resulting from not having access to the implant, the following calculations were utilized:

1. To estimate the number of potential unintended pregnancies resulting from the use of any contraceptive method (*UP*_*x*_), the failure rate of that contraceptive method is multiplied by the number of women using it:
  $$\begin{array}{c} UP_{x}=failurerateofmethodx\left(FR_{x}\right)*numberofwomen\\ choosingmethodxifimplantnotavailable \end{array}$$
2. Number of potential unintended pregnancies if the implant is available (*UP_A_*)
  To estimate (*UP_A_*), the failure rate of the most effective contraceptive method currently used is multiplied by the number of women currently using it. Because respondents were allowed to select multiple types of contraceptives, a conservative approach was taken and the contraceptive method with the lowest failure rate reported by the respondent was considered for the calculation of the estimated number of unintended pregnancies.
  $$\begin{array}{c} UP_{A}=failurerateofmethodx\left(FR_{x}\right)*numberofwomen\\ choosingmethodxifimplantisavailable \end{array}$$
3. Number of potential unintended pregnancies when the implant is not available
  Women who are currently using the implant, and those who would be willing to switch to an implant, were asked what their second contraceptive method of choice would be should the implant not be available. As earlier, because respondents were allowed to select multiple types of contraceptives if the implant was unavailable, a conservative approach was taken and the contraceptive method with the lowest failure rate reported by the respondent was considered for the calculation of the estimated number of unintended pregnancies.
  To estimate *UP_NA_*, the failure rate of the most effective second choice contraceptive method was multiplied by the number of women who are currently using or would be willing to use an implant and have chosen that method.
  $$\begin{array}{c} UP_{NA}=failurerateofmethodx\left(FR_{x}\right)*numberofwomen\\ choosingmethodxifimplantisnotavailable \end{array}$$
4. To estimate the total number of potential unintended pregnancies due to making the implant unavailable (*UPT*), the number of potential unintended pregnancies if the implant is available (*UP_A_*) was subtracted from the number of potential unintended pregnancies when the implant is not available (*UP_NA_*):
  $$UPT=UP_{NA}-UP_{A}$$
  All analyses were stratified by age groups. Frequencies and proportions are reported for unweighted and weighted data.

## Results

### Participant characteristics

The mean age for the total sample of 1,200 women was 30.42 ± 7.67 years. Most participants were white (69%), not of Hispanic, Latino, or Spanish origin (84%), and about half of all participants were single and had never been married (48%). The educational level, employment status, and household income varied among participants, with 39% having earned a high school degree or equivalent, 44% having a full-time job, and 27% an annual income between $25,000 and $50,000. The majority of participants had commercial health insurance (64%). The average BMI of the total sample was 26.36 ± 6.92 (47%) (Table 1).

**Table 1.** Sociodemographic Characteristics per Age Group

	Age: 18–24 *n* = 400	Age: 25–34 *n* = 400	Age: 35–44 *n* = 400	Total *n* = 1,200
Age				
Mean ± SD	21.73 ± 1.71	30.05 ± 2.79	39.48 ± 2.83	30.42 ± 7.67
95% CI	21.56–21.89	29.77–30.32	39.21–39.76	29.98–30.85
Race, *n* (%)				
White	221 (55%)	302 (76%)	302 (76%)	825 (69%)
Black/African American	78 (20%)	44 (11%)	47 (12%)	169 (14%)
Other	98 (24%)	53 (13%)	51 (13%)	202 (17%)
Decline to answer	3 (1%)	1 (0%)	0 (0%)	4 (0%)
Ethnicity, *n* (%)				
Not of Hispanic, Latino, or Spanish origin	305 (76%)	341 (85%)	357 (89%)	1,003 (84%)
Mexican, Mexican American, Chicano/Puerto Rican/Cuban/Other Hispanic Latino, or Spanish origin	93 (23%)	59 (15%)	43 (11%)	195 (16%)
Decline to answer	2 (0%)	0 (0%)	0 (0%)	2 (0%)
Marital status, *n* (%)				
Married/living with a partner	102 (26%)	198 (50%)	258 (64%)	558 (46%)
Single, never married	292 (73%)	185 (46%)	104 (26%)	581 (48%)
Others (divorced, separated, widowed)	5 (0%)	17 (4%)	36 (9%)	58 (4%)
Decline to answer	1 (0%)	0 (0%)	2 (0%)	3 (0%)
Education, *n* (%)				
Less than high school or equivalent	41 (10%)	17 (4%)	12 (3%)	70 (6%)
High school or equivalent	247 (62%)	113 (28%)	108 (27%)	468 (39%)
Associate's degree	26 (6%)	39 (10%)	60 (15%)	125 (10%)
Bachelor's degree	75 (19%)	172 (43%)	140 (35%)	387 (32%)
Graduate degree	8 (2%)	59 (15%)	80 (20%)	147 (12%)
Decline to answer	3 (1%)	0 (0%)	0 (0%)	3 (0%)
Employment status, *n* (%)				
Employed (full-, part-time, self-employed)	187 (48%)	285 (70%)	269 (66%)	741 (62%)
Homemaker/retired	29 (7%)	60 (15%)	94 (24%)	183 (16%)
Student	126 (32%)	22 (6%)	5 (1%)	153 (13%)
Disability	19 (5%)	25 (6%)	19 (5%)	63 (5%)
Unemployed	41 (9%)	7 (1%)	13 (3%)	59 (4%)
Decline to answer	0 (0%)	1 (0%)	0 (0%)	1 (0%)
Household income, *n* (%)				
<$25,000	145 (36%)	61 (15%)	52 (13%)	258 (22%)
$25,000 to <$50,000	103 (26%)	133 (33%)	91 (23%)	327 (27%)
$50,000 to <$75,000	55 (14%)	80 (20%)	105 (26%)	240 (20%)
$75,000 or more	68 (17%)	121 (31%)	138 (35%)	327 (27%)
Decline to answer	29 (7%)	5 (1%)	14 (3%)	48 (4%)
Insurance status, *n* (%)				
Insured: commercial	217 (54%)	273 (68%)	282 (70%)	772 (64%)
Insured: Medicare	30 (8%)	14 (4%)	13 (3%)	57 (5%)
Insured: Medicaid	69 (17%)	56 (14%)	53 (13%)	178 (15%)
Insured: other (*i.e.*, VA/CHAMPUS, TRICARE, not sure)	10 (2%)	8 (2%)	9 (2%)	27 (2%)
Not insured	63 (16%)	48 (12%)	43 (11%)	154 (13%)
BMI (continuous)				
Mean ± SD	25.87 ± 6.88	25.90 ± 6.63	27.32 ± 7.14	26.36 ± 6.92
95% CI	25.18–26.56	25.24–26.57	26.61–28.04	26.96–26.76

BMI, body mass index; CI, confidence interval.

### Contraceptive use

Women were asked which method or methods of contraception they were using at the time of the study, and a majority (88%) reported using at least one form of contraceptive.

**FIG. 1.**
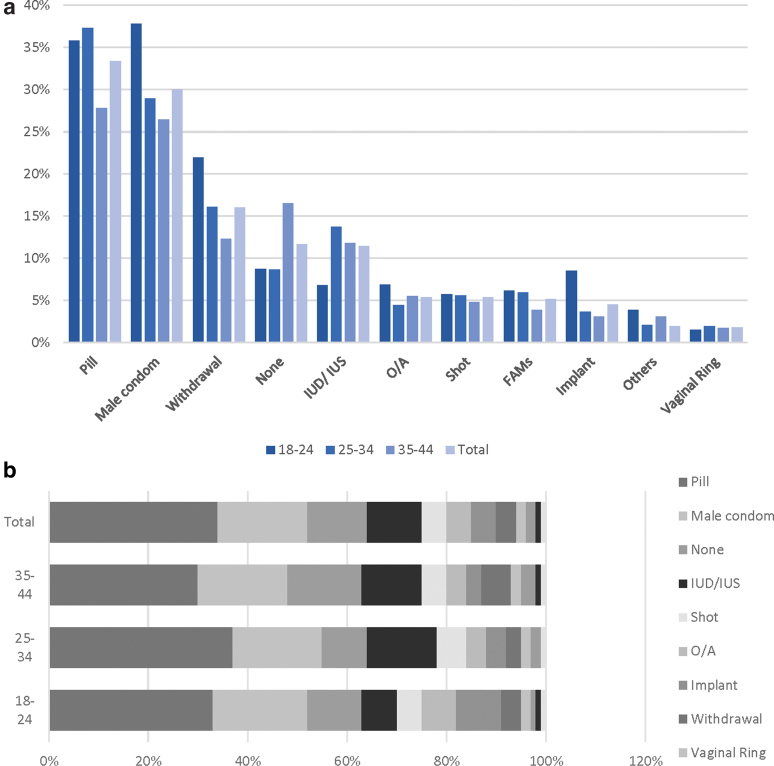
**(a)** Proportions of current contraceptive use per age group (all contraceptives; weighted estimates). Others: patch, breastfeeding, diaphragm, female condom, cervical cap, spermicide. **(b)** Proportions of current most effective contraceptive use per age group (weighted estimates). FAMs, fertility awareness methods; IUD, intrauterine device; IUS, intrauterine system; O/A, outercourse and abstinence.

Figure 1a illustrates all the contraceptive methods reported by women. The most commonly used across all age groups were the contraceptive pill and the male condom, used by 36% and 38% of the women in the 18–24 age group, respectively, 37% and 29% in the 25–34 age group, and 28% and 26% in the 35–44 age group. Among the LARCs, the IUD/IUS were the most commonly used, by 11% of the women, with the largest group of users among the 25–34 years old age group The group of women most using the implant were between 18 and 24 years old (9%) (Fig. 1a).

Figure 1b illustrates only the most effective method chosen by women. Across all age groups, the most effective method women reported using was the contraceptive pill (30%–37%), followed by the male condom.

### Willingness to switch to another form of birth control

**FIG. 2.**
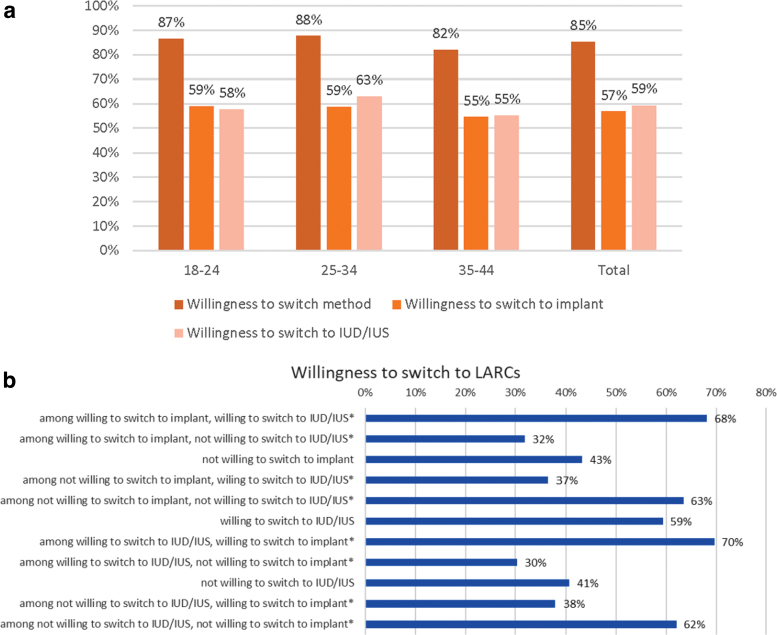
**(a)** Willingness to switch contraceptive (weighted). **(b)** Willingness to switch stratified by willingness to switch to IUD/IUS (no/yes) and willingness to switch to implant (no/yes) (weighted). *Data presented are not meant to sum to 100%. The graph represents the cross tabulation results of those willing and not willing to switch from one form of LARC to the other. LARC, long-acting reversible contraceptive method.

The weighted estimates indicate that most women in the U.S. population (85%) would report being open to switching to a different form of birth control should it be freely available with no access or cost restraints. Willingness to switch was very similar across age groups. In regard to switching to LARCs specifically, more than half the women indicated that they would be willing to switch to an arm implant (between 55% and 59%, across age groups). Also, more than half of women indicated they would be willing to try an IUD/IUS (55%–63%, across age groups), should these options be freely available to them (Fig. 2a).

To understand the population of women who could potentially be excluded from, or experience barriers to, LARC use, should the implant be unavailable, the percentage of women willing to switch to an implant but not an IUD/IUS were examined (Fig. 2b). Among those women not willing to switch to an IUD/IUS, weighted estimates showed approximately a third (38%) were willing to switch to the arm implant. Conversely, among women willing to switch to an implant, 32% were not willing to switch to an IUD/IUS.

### Reasons for not considering an IUD/IUS

**FIG. 3.**
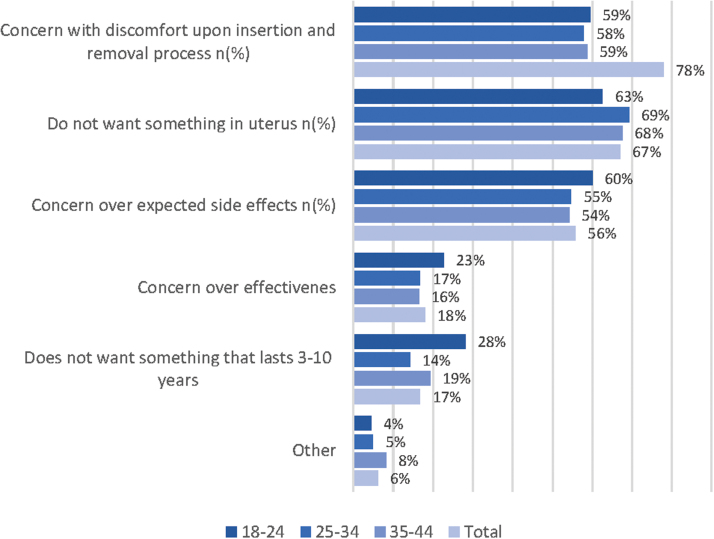
Reasons for not considering IUD.

The most commonly selected reasons for not considering an IUD/IUS among women who reported they were unwilling to switch to this form of birth control included concern with discomfort with insertion/removal process (78%), not wanting something in their uterus (67%), and concern over expected side effects (56%) (Fig. 3 and Supplementary Table S1).

### Second choice of contraception should the implant not be available

**FIG. 4.**
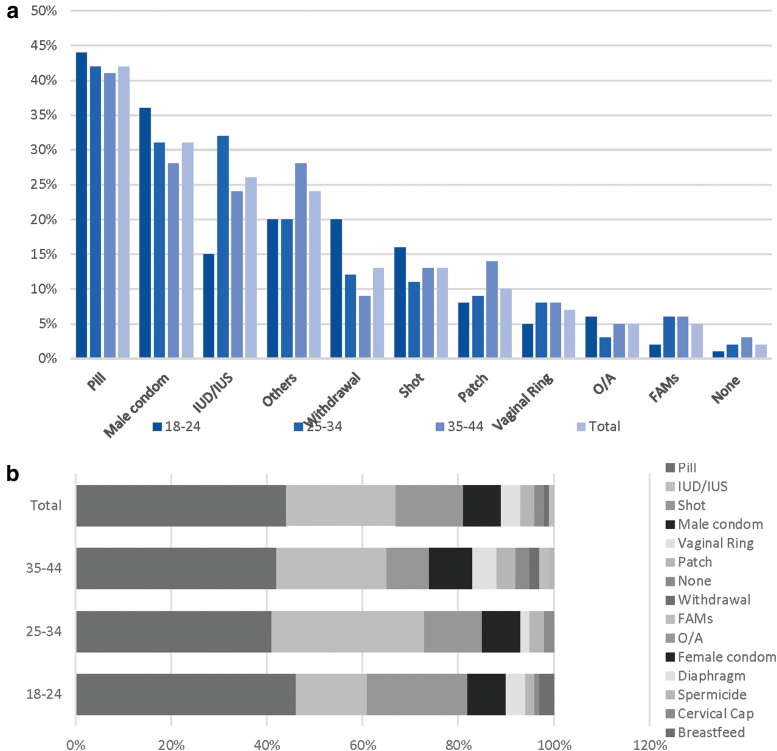
**(a)** Second choices if implant is not available, multiple responses allowed (weighted). Others: patch, breastfeeding, diaphragm, female condom, cervical cap, spermicide. **(b)** Proportions of second choice of contraceptive if implant is not available, most effective form only, per age group (weighted estimates).

Among participants who reported that they are open to the implant, or are currently using it, 42% reported that their second choice of contraception, should the implant not be available, would be the birth control pill across all age groups. However, this second choice for a contraceptive varied to some extent. Despite all age groups having a stronger preference for oral contraceptives, the older age group 35–44 preferred the male condom (28%) to the IUD/IUS (24%), whereas the 25–34 years old had a similar preference for these two types of contraception (31% and 32%, respectively) (Fig. 4a).

If the implant would not be available, the pill was the preferred most effective contraception method across all age groups, with 44% of the total women population opting for this method, with or without concomitant use of other methods. The second most effective choice was the IUD/IUS in the 25–34 (32%) and 35–44 (23%) age groups, whereas the 18–24 year olds preferred the quarterly shot (Fig. 4b).

### Prevalence of unintended pregnancies

The number of women aged 18–44 years old in the United States who could potentially be underserved by not being provided equal access to the subdermal implant, that is, the total number of current implant users and the nonimplant users who would be willing to switch to an implant, was calculated to be 20,562,118 out of an estimated weighted population of 37,965,109, representing 54% of women.

The proportion of women in the United States who would potentially be underserved by not being provided equal access to the subdermal implant contraceptive was assessed by combining the proportion of women who were currently using a subdermal implant (1,702,998/37,965,109; 4%) and the proportion of women with some degree of willingness to switch to an implant (18,859,120/37,965,109; 50%).

The prevalence of potential unintended pregnancies resulting from making the implant unavailable was estimated to be 8% among the total sample. The prevalence was slightly higher among women in the oldest age group compared with younger women (7% for women aged 18–24 years, 7% for women aged 25–34 years, and 9% for women aged 35–44 years). The number and proportion of unintended pregnancies for implant users and for nonimplant users, as well as the total number of unintended pregnancies due to making the implant unavailable per age group amounts to a total of 1,597,315 (Supplementary Table S2).

## Discussion

Overall, the majority of women reported that they would be willing to switch to a LARC, should it be readily available to them. Willingness to switch to a LARC was similar for both the IUD/IUS and the implant across all age groups, ranging between 55% and 63%. It was, however, observed that current use of IUDs is in general higher than the use of implants, with the exception of 18–24 years old women.

Lower use of implants compared with IUDs among LARC users could be due to either patient preference or access, and this study can offer preliminary insight about women's preferences without the constraint of access. Although the ACA and follow-up HHS guidance require first dollar coverage for contraceptive care, other clinical factors may affect access to LARC options.^18^ These results suggest that women should be given equal access to all forms of contraception.

A significant proportion of women, nearly half (41%), would not be willing to switch to an IUD mostly due to feelings about or experiences with insertion and removal of the device, not wanting something in their uterus or concerns with side effects. Moreover, similar findings were observed across age groups.

However, among those not willing to switch to an IUD/IUS, 38% would be willing to switch to an implant and among those willing to switch to an implant, 32% would not be willing to switch to an IUD/IUS. These estimates suggest these women potentially could be underserved should the implant be made unavailable by limiting access to their LARC of reported preference. It would, therefore, be particularly important for women across all age groups to be able to be provided with equal access to another type of LARC that would ensure a higher effective protection against unintended pregnancies. Our results support previous findings, as we observe that, if the implant was unavailable, there may be an increase in the number of pregnancies.

Limitations in this study, as in other surveys, could include errors related to missing responses that could affect the results. Our programming made use of quality control procedures to eliminate the risk for missing responses and thus the effects of such errors.

Under-reporting of sensitive behaviors might have affected the results of the survey. The use of an anonymous self-reported electronic survey minimized this issue. An overestimation of the number of unintended pregnancies could have been made, as not all women reporting willingness to switch their current contraceptive method to an implant would actually follow through with utilization; respondents were not asked about their experiences with contraceptive counseling and may have already had discussed their contraceptive needs with their providers.

In contrast, for those women who reported multiple current methods of contraception, only the one with the lowest failure rate was used to estimate the number of unintended pregnancies due to lack of access to the implant. This methodology provides a conservative estimate, because respondents could be using those methods either simultaneously or separately.

For those who use them separately, the potential number of unintended pregnancies would have been underestimated in this study, as we are not considering for the calculation the highest failure rate possible for that given respondent. No adjustments were made for contraceptive discontinuation rates and, therefore, the calculation of unintended pregnancies provides a rough, and potentially upper limit, estimate. Given the nature of this sample survey, persons who are incarcerated or otherwise institutionalized, homeless, those living on military bases in the United States, those who are not able to write and read in English, and those without access to an internet-based survey were excluded.

These individuals may have different patterns of sexual behavior, and results cannot, therefore, be generalized to those populations. Characteristics, including representativeness, and limitations of the NHWS have been widely published.^19–21^ In addition, sexual behavior and contraceptive choices may be a sensitive topic; potential respondents who were not willing to share this information will not be represented in the data, and this limitation would likely affect estimates.

## Conclusions

Overall, the majority of women (72.6%) reported that they would be willing to switch to a LARC, should it be readily available to them. Considering those women who already use an implant and those who would be willing to switch to it, more than half of all women (58%) would be underserved by not being provided equal access to the subdermal implant. This reduced availability of this type of LARC may alone elevate the number of unintended pregnancies in the United States by ∼1,597,315 pregnancies per year, representing 8% of all pregnancies.

Therefore, it is important to make all the contraceptive forms available and to maintain access to both types of LARCs (IUDs/IUSs and subdermal implant) in the United States, to help reduce unintended pregnancies and better serve women and their family planning needs.

## Supplementary Material

Supplemental data

Supplemental data

Supplemental data

## Abbreviations Used

ACOG: American College of Obstetricians and Gynecologists
AAP: American Academy of Pediatrics
BMI: body mass index
CI: confidence interval
FAMs: fertility awareness methods
HHS: U.S. Department of Health and Human Services
IUD: intrauterine devices
IUS: intrauterine systems
LARCs: long-acting reversible contraceptive methods
NHWS: U.S. National Health Wellness Survey
NQF: National Quality Forum
NSFG: National Survey of Family Growth
O/A: outercourse and abstinence
ob/gyns: obstetricians and gynecologists
