# Supplementary material for: Preference for Contraceptive Implant Among Women 18–44 years old
Source: Womens Health Rep (New Rochelle). 2021 Dec 15;2(1):622–32. doi: 10.1089/whr.2021.0113 (PMC8820401; doi:10.1089/whr.2021.0113)
Supplement: Supplemental data [file Suppl_Data.docx]

**Notice of Confidentiality**

The following material is not generally available to the public and is proprietary to Kantar Health. It shall be kept confidential by the Receiving Party and not disclosed to any third party without the written permission of Kantar Health.

**KH 161103542-1**

IC. Do you agree to participate (take part) in this survey?

Choose one response.

| 1 | Yes, I agree to participate |
| --- | --- |
| 2 | No, I do not agree to participate |

S1. Please indicate your sex.

SELECT ONLY ONE

| Male | 1 |
| --- | --- |
| Female | 2 |

S2. What is your year of birth? *Please enter as a four-digit number, e.g., 1963*.

|__|__|__|__| **[RANGE 1900- 2014]**

S3. Are you currently pregnant, or trying to get pregnant?

SELECT ONLY ONE.

| Yes, trying to get pregnant | 1 |
| --- | --- |
| Yes, currently pregnant | 2 |
| No, not trying to get pregnant | 3 |
| Prefer not to say | 4 |

S4. Are you currently, or plan to be, sexually active with a male….?

SELECT ONLY ONE.

| Yes | 1 |
| --- | --- |
| No | 2 |

S5. Have you ever had a…?

| Hysterectomy (surgical removal of uterus) | 1 |
| --- | --- |
| Bilateral salpingo-oophorectomy (BSO) (surgical removal of both ovaries) | 2 |
| Tubal ligation (tubes tied/sterilization) | 3 |
| General infertility (doctor has told you cannot get pregnant) | 4 |
| Committed/lifelong male partner who has undergone a vasectomy | 5 |
| None of the above | 6 |

FOR S7 SHOW CARDS ONE AT A TIME

S7. What contraceptive method have you ever used? Please take a moment to review the chart completely.

SELECT ALL THAT APPLY

S7A. What contraceptive method(s) are you currently using? Please take a moment to review the chart completely.

SELECT ALL THAT APPLY

| 1 | | 2 | 3 | 4 |
| --- | --- | --- | --- | --- |
| 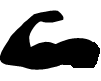 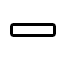  ***Birth control implant***  *This is a small, thin rod about the size of a matchstick. The implant releases hormones into your body that prevent you from getting pregnant. A nurse or doctor places the implant under your skin on your upper arm.*  *.*  Lasts up to 3 years  Brand Names Include: Nexplanon, Implanon | | **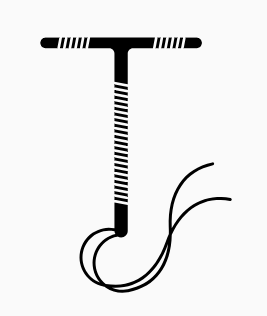**  **IUD/IUS**  *This is a small device that's put into your uterus to prevent pregnancy. There are non-hormonal options or copper (non-hormonal).*  Lasts up to 3-6 years  Brand Names Include: ParaGard (Copper), Mirena (hormonal), Kyleena, (hormonal) Liletta (hormonal), and Skyla (hormonal). | 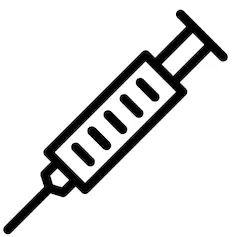  **Birth Control Shot**  *This is an injection you get from a nurse or doctor once every 3 months.*  Get every 3 months  Brand Names Include: Depo-Provera (also called the Depo shot, or DMPA) | 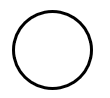  **Birth Control Vaginal Ring**  *This is a birth control ring that you place inside your vagina.*  Monthly  Brand Names Include: NuvaRing |
| 5 | | 6 | 7 | 8 |
| 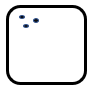  **Birth Control patch**  *This is a small patch that you wear on the skin of your belly, upper arm, butt, or back. A new patch gets placed every week for 3 weeks, and it releases hormones that prevent pregnancy. Then you get a week off before you repeat the cycle.*  Replace weekly  Brand Names Include: Xulane | | 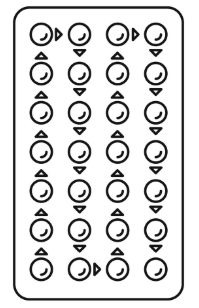  **Birth Control Pill**  *These are a kind of medicine (pill or mini-pill) with hormones that you take every day to prevent pregnancy. Some pills have a combination of estrogen/progesterone, some are progesterone only, and some offer an extended cycle (so you menstruate less frequently). You are responsible for taking your pill daily.*  Take daily  Brand Names Include: Mircette, Aviane, Natazia, Estrostep, Levora, Levlite, Lessina, Enpresse, Aranelle, Lo/ovral-28, Ortho Tri-Cyclen, Ortho-Novum, Alesse, Levlen, Loestrin, Apri, Yaz, Yasmin, Nordette, Micronor, Camila, Errin, Jolivette, Seasonale, Lybrel, Quasense, Jolessa, Seasonique | 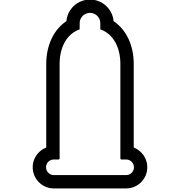  **Male Condom**  *Condoms are thin, stretchy pouches that a man wears on his penis during sex. A new condom must be used each time you have sex. These also protect against STIs/STDs*  Use every time  Brand Names Include: Durex, Lifestyles, Trojan, Crown, Atlas, Kimono | 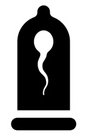  **Female Condom**  *Female condoms go inside your vagina for pregnancy prevention or into the vagina for protection from STDs. They’re sometimes called internal condoms.*    Use every time  Brand Names Include: FC2 Female Condom |
| 9 | 11 | | 12 |  |
| 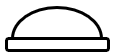  **Diaphragm**  *This is a shallow, bendable cup that you put inside your vagina. You bend it in half and insert it inside your vagina to cover your cervix. It covers your cervix during sex to prevent pregnancy.* *In order for a diaphragm to work best, it must be used with spermicide. It must be put in prior to sex.*  Use every time | 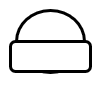  **Cervical Cap**  *This is a little cup made from soft silicone. You put it deep inside your vagina to cover your cervix. It works by stopping sperm from joining an egg. For a cervical cap to work best, it must be used with spermicide.*  Use every time  Brand Names Include: FemCap | | 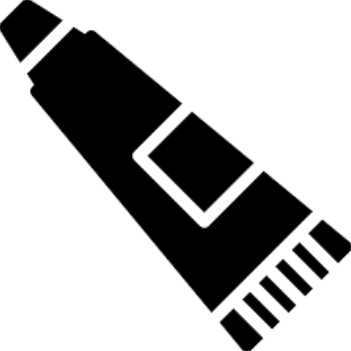  **Spermicide**  *Spermicide contains chemicals that stop sperm from reaching an egg. You put it in your vagina before sex.* *Spermicide can be used by itself or combined with other birth control methods. Formulation come in different forms such as gel, foam, cream, film or suppository.*  Use every time |  |
| 13 | 14 | | 15 | 16 |
| 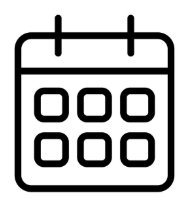  **Fertility Awareness (FAMs)**  *Fertility awareness methods (FAMs) are ways to track your ovulation so you can prevent pregnancy. Also called “Rhythm/calendar method” and "natural family planning”.* Y*ou track your menstrual cycle, so you’ll know when your ovaries release an egg every month. The days near ovulation fertile)- you are likely to get pregnant. To prevent pregnancy, you refrain from sex or use another birth control method (like condoms) on those “unsafe,” fertile days. Some women use a special thermometer/app to help track ovulation days.*  Use daily | 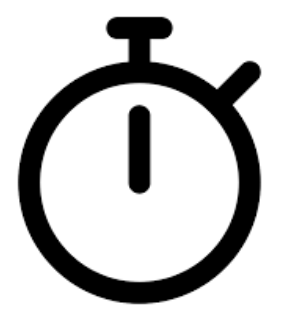  **Withdrawal**  **(Pull-Out Method)**  *Pulling out (also known as withdrawal) is a way to prevent pregnancy by keeping semen away from the vagina. pulling the penis out of the vagina before ejaculation.*  Use every time | | 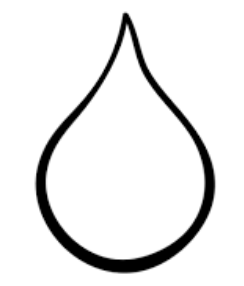  **Breastfeeding as Birth Control**  *When you exclusively breastfeed (nursing at least every 4 hours during the day and every 6 hours at night) — your body naturally stops ovulating. You can’t get pregnant if you don’t ovulate.* *No ovulation means you won’t have your period, either.*  Breastfeed every 4-5 hrs. | 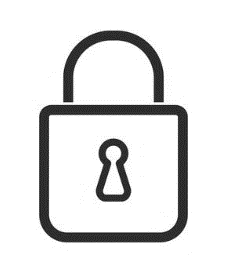  **Outercourse & Abstinence**  *The definition of abstinence is when you don’t have sex. Outercourse is other sexual activities besides vaginal sex.*  Used every time |
| 99 |  |  |  |  |
| 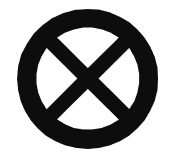  **None of these contraception options are used.**  **[EXCLUSIVE]** |  |  |  |  |

S9. Thank you for your response. You are eligible to take part in this survey. It should take less than 15 minutes to complete. You may quit the survey by simply closing out the window, at any time. If you complete the full survey you get **[50 life points]** for your participation.

We’ll be asking about family planning and your preferred methods of contraception and why. This survey is being taken by 1,200 women, just like you, across the US. The results of this survey may be used in medical and scientific literature to help inform doctors, insurers, and policy makers on the preference women have when it comes to choice of contraception.

If you’re interested click “I’m in, take me to the survey”.

SELECT ONLY ONE.

| I’m in, take me to the survey | 1 |
| --- | --- |
| No thanks, not interested | 2 |

A100. Overall, how willing/open would you be to switch to a different birth control method if it were freely available with no access or cost constraints?

| **Not at all willing to switch** | **Somewhat willing to switch** | **Moderately willing to switch** | **Very willing to switch** | **Extremely willing to switch** |
| --- | --- | --- | --- | --- |
| 1 | 2 | 3 | 4 | 5 |

A105. Given the information below, how willing/open would you be to switch to **[IF BOTH OPTIONS SHOWN:** **each** of**]** the long acting reversible contraception options (LARC) below should they be freely available with no access or cost constraints? As a reminder, the information for these options are shown below.

| 1 | 2 |
| --- | --- |
| 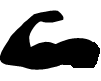 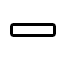  ***Birth control implant***  *This is a small, thin rod about the size of a matchstick. The implant releases hormones into your body that prevent you from getting pregnant. A nurse or doctor places the implant under your skin on your upper arm.*  0.1% rate of failure as commonly used **[PN: HOVER DEFINITION]**  Lasts up to 3 years  Brand Names Include: Nexplanon, Implanon | 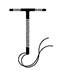 **IUD/IUS**  *This is a small device that's put into your uterus to prevent pregnancy. There are non-hormonal options or copper (non-hormonal).*  0.7-0.8% rate of failure as commonly used **[PN: HOVER DEFINITION]**  Lasts up to 3-10 years  Brand Names Include: ParaGard (Copper), Mirena (hormonal), Kyleena, (hormonal) Liletta (hormonal), and Skyla (hormonal). |

**[PN: HOVER DEFINITION** percent of women who are estimated to become pregnant by the end of 1 year of typical use.**]**

| **Not at all willing to switch** | **Somewhat willing to switch** | **Moderately willing to switch** | **Very willing to switch** | **Extremely willing to switch** |
| --- | --- | --- | --- | --- |
| 1 | 2 | 3 | 4 | 5 |

A110. From the reasons below, please select those that are most similar to yours, as to why you would not consider an IUD/IUS.

|  | IUDs (such as ParaGard (Copper))  IUSs (such as Mirena, Kyleena, Liletta, and Skyla) |
| --- | --- |
| 1 | I do not want something in my uterus |
| 2 | Concern over effectiveness |
| 3 | Concern with discomfort with insertion/removal process |
| 4 | Concern over potential side effects |
| 5 | Don't want something that lasts up to 3-10 years |
| 6 | Other |

A115. Now let’s assume there is **no** option for you to obtain a birth control implant (such as, it is not available from pharmacy or insurance). In this circumstance which method of contraception would you prefer from the following options it they were freely available with no access or cost constraints.

| 1 | 2 | 3 | 4 |
| --- | --- | --- | --- |
| ***Birth control implant-***  ***NOT AVAILABLE*** | 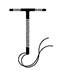 **IUD/IUS**  *This is a small device that's put into your uterus to prevent pregnancy. There are non-hormonal options or copper (non-hormonal).*  0. 7-0.8% rate of failure as commonly used **[PN: HOVER DEFINITION]**  Lasts up to 3-6 years  Brand Names Include: ParaGard (Copper), Mirena (hormonal), Kyleena, (hormonal) Liletta (hormonal), and Skyla (hormonal).  **IUD/IUS*-***  ***NOT AVALIABLE*** | 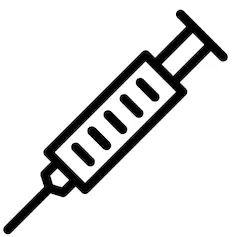  **Birth Control Shot**  *This is an injection you get from a nurse or doctor once every 3 months.*  3-4% rate of failure as commonly used **[PN: HOVER DEFINITION]**  Get every 3 months  Brand Names Include: Depo-Provera (also called the Depo shot, or DMPA) | 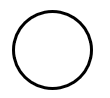  **Birth Control Vaginal Ring**  *This is a birth control ring that you place inside your vagina.*  ~~9~~7% rate of failure as commonly used**[PN: HOVER DEFINITION]**  Monthly  Brand Names Include: NuvaRing |
| 5 | 6 | 7 | 8 |
| 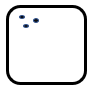  **Birth control patch**  *This is a small patch that you wear on the skin of your belly, upper arm, butt, or back. A new patch gets placed every week for 3 weeks, and it releases hormones that prevent pregnancy. Then you get a week off before you repeat the cycle.*  7% rate of failure as commonly used**[PN: HOVER DEFINITION]**  Replace weekly  Brand Names Include: Xulane | 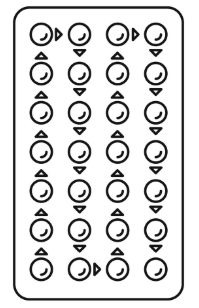  **Birth Control Pill**  *These are a kind of medicine (pill or mini-pill) with hormones that you take every day to prevent pregnancy. Some pills have a combination of estrogen/progesterone, some are progesterone only, and some offer an extended cycle (so you menstruate less frequently). You are responsible for taking your pill daily.*  7% rate of failure as commonly used **[PN: HOVER DEFINITION]**  Take daily  Brand Names Include: Mircette, Aviane, Natazia, Estrostep, Levora, Levlite, Lessina, Enpresse, Aranelle, Lo/ovral-28, Ortho Tri-Cyclen, Ortho-Novum, Alesse, Levlen, Loestrin, Apri, Yaz, Yasmin, Nordette, Micronor, Camila, Errin, Jolivette, Seasonale, Lybrel, Quasense, Jolessa, Seasonique | 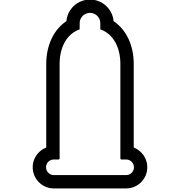  **Male Condom**  *Condoms are thin, stretchy pouches that a man wears on his penis during sex. A new condom must be used each time you have sex. These also protect against STIs/STDs*  13% rate of failure as commonly used **[PN: HOVER DEFINITION]**  Use every time  Brand Names Include: Durex, Lifestyles, Trojan, Crown, Atlas, Kimono | 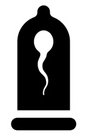  **Female Condom**  *Female condoms go inside your vagina for pregnancy prevention or into the vagina for protection from STDs. They’re sometimes called internal condoms.*    21% rate of failure as commonly used **[PN: HOVER DEFINITION]**  Use every time  Brand Names Include: FC2 Female Condom |

| 9 | 11 | 12 | | | |  |
| --- | --- | --- | --- | --- | --- | --- |
| 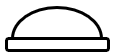  **Diaphragm**  *This is a shallow, bendable cup that you put inside your vagina. You bend it in half and insert it inside your vagina to cover your cervix. It covers your cervix during sex to prevent pregnancy.* *In order for a diaphragm to work best, it must be used with spermicide. It must be put in prior to sex.*  17% rate of failure as commonly used **[PN: HOVER DEFINITION]**  Use every time | 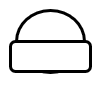  **Cervical Cap**  *This is a little cup made from soft silicone. You put it deep inside your vagina to cover your cervix. It works by stopping sperm from joining an egg. For a cervical cap to work best, it must be used with spermicide.*  16-32% rate of failure as commonly used**[PN: HOVER DEFINITION]**  Use every time  Brand Names Include: FemCap | 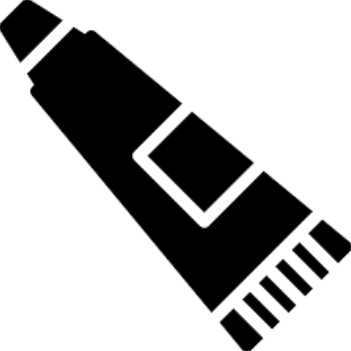  **Spermicide**  *Spermicide contains chemicals that stop sperm from reaching an egg. You put it in your vagina before sex.* *Spermicide can be used by itself or combined with other birth control methods. It does not “kill” sperm, but it slows it down, so it can’t reach an egg to prevent pregnancy.*  21% rate of failure as commonly used **[PN: HOVER DEFINITION]**  Use every time | | | |  |
| 13 | 14 | | 15 | | 16 | |
| 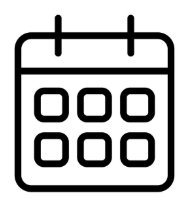  **Fertility Awareness (FAMs)**  *Fertility awareness methods (FAMs) are ways to track your ovulation so you can prevent pregnancy. Also called “Rhythm/calendar method” and "natural family planning”.* Y*ou track your menstrual cycle, so you’ll know when your ovaries release an egg every month. The days near ovulation fertile)- you are likely to get pregnant. To prevent pregnancy, you refrain from sex or use another birth control method (like condoms) on those “unsafe,” fertile days. Some woman use a special thermometer/app to help track ovulation days.*  12-23% rate of failure as commonly used**[PN: HOVER DEFINITION]**  Use daily | 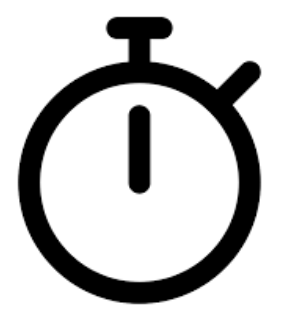  **Withdrawal**  **(Pull-Out Method)**  *Pulling out (also known as withdrawal) is a way to prevent pregnancy by keeping semen away from the vagina. pulling the penis out of the vagina before ejaculation.*  20% rate of failure as commonly used **[PN: HOVER DEFINITION]**  Use every time | | 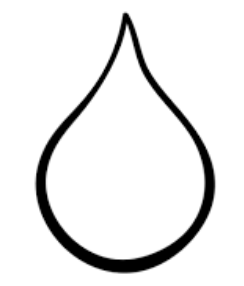  **Breastfeeding as Birth Control**  *When you exclusively breastfeed (nursing at least every 4 hours during the day and every 6 hours at night) — your body naturally stops ovulating. You can’t get pregnant if you don’t ovulate.* *No ovulation means you won’t have your period, either.*  2% rate of failure as commonly used**[PN: HOVER DEFINITION]**  Breastfeed every 4-5 hrs. | | 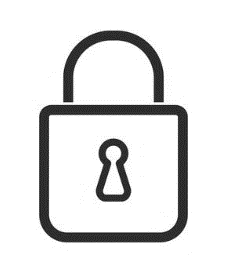  **Outercourse & Abstinence**  *The definition of abstinence is when you don’t have sex. Outercourse is other sexual activities besides vaginal sex.*  No established pregnancy rate with typical use (100% efficacy when practiced perfectly & correctly every time)  Used every time | |
| 99 |  | | |  |  |  |
| 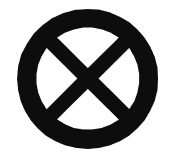  **None of these contraception options.**  **[EXCLUSIVE]** |  | | |  |  |  |

**CLASSIFICATION**

To finish up, we have a couple classification questions.

C110. What is your height?

|_| feet |_|_| inches

**RANGE: 3-7 RANGE: 0-11 INCHES**

C115. What is your weight?

|_|_|_| lbs. **RANGE = 80 – 600 lbs**

99 Decline to answer

C119. Are you of Hispanic, Latino, or Spanish origin?

| No, not of Hispanic, Latino, or Spanish origin | 1 |
| --- | --- |
| Yes, Mexican, Mexican American, Chicano | 2 |
| Yes, Puerto Rican | 3 |
| Yes, Cuban | 4 |
| Yes, other Hispanic, Latino, or Spanish origin (for example, Argentinean, Colombian, Dominican, Nicaraguan, Salvadorian, Spaniard, and so on) | 5 |
| Decline to answer | 99 |

C120. What is your race?

| White | 1 |
| --- | --- |
| Black, African American, or Negro | 2 |
| Asian, Asian American (for example Asian Indian, Chinese, Filipino, Japanese, Korean, Vietnamese) | 3 |
| Native Hawaiian or Other Pacific Islander | 4 |
| American Indian or Alaskan native | 5 |
| Some other race or origin | 6 |
| Decline to answer | 99 |

C125. What is your marital status?

| Married | 1 |
| --- | --- |
| Single, never married | 2 |
| Divorced | 3 |
| Separated | 4 |
| Widowed | 5 |
| Living with partner | 6 |
| Decline to answer | 99 |

C130. What is the highest level of education you have completed or the highest degree you have received?

| Less than high school | 1 |
| --- | --- |
| Completed some high school | 2 |
| High school graduate or equivalent (e.g., GED) | 3 |
| Completed some college, but no degree | 4 |
| Associate’s degree | 5 |
| College graduate (e.g., B.A., A.B., B.S.) | 6 |
| Completed some graduate school, but no degree | 7 |
| Completed graduate school (e.g., M.S., M.D., Ph.D.) | 8 |
| Decline to answer | 99 |

C135. Which of the following income categories best describes your total [CURRENT YEAR – 1] household income before taxes?

| Less than $15,000 | 1 |
| --- | --- |
| $15,000 to $24,999 | 2 |
| $25,000 to $34,999 | 3 |
| $35,000 to $49,999 | 4 |
| $50,000 to $74,999 | 5 |
| $75,000 to $99,999 | 6 |
| $100,000 to $124,999 | 7 |
| $125,000 to $149,999 | 8 |
| $150,000 to $199,999 | 9 |
| $200,000 to $249,999 | 10 |
| $250,000 or more | 11 |
| Decline to answer | 99 |

C140. Including you, how many adults (age 18 or over) live in your household?

|_|_| PROGRAMMER: **RANGE =1-20**

C145. How many children under the age of 18 live in your household?

|_|_| PROGRAMMER: **RANGE=0-15**

C150. Do you currently have health insurance?

| Yes | 1 |
| --- | --- |
| No | 2 |

C155. What kind of health insurance do you have as your primary insurance?

| Insurance coverage through a current or former employer | 1 |
| --- | --- |
| Insurance coverage through spouse's/partner’s employer | 2 |
| Individual/Family insurance plan thru a State Health Exchange | 3 |
| Individual/Family insurance plan purchased directly by you | 4 |
| Medicaid (MediCal for California residents) | 5 |
| Medicare | 6 |
| Veterans administration (VA)/CHAMPUS | 7 |
| TRICARE | 8 |
| Insurance coverage through my parent’s or legal guardian’s employer | 9 |
| Not sure | 10 |
| Decline to answer | 99 |

C160. You indicated that you have Medicare health insurance. Which of the following types of Medicare do you currently have?

| Medicare Part A | 1 |
| --- | --- |
| Medicare Part B | 2 |
| Medicare Part C/Advantage with prescription drug coverage | 3 |
| Medicare Part C/Advantage without prescription drug coverage | 4 |
| Medicare Part D | 5 |
| Decline to answer | 99 |

C165. What is your employment status?

| Employed full time | 1 |
| --- | --- |
| Self-employed | 2 |
| Employed part time | 3 |
| Homemaker | 4 |
| Retired | 5 |
| Student | 6 |
| Long-Term Disability | 7 |
| Short-Term Disability | 8 |
| Not employed, but looking for work | 9 |
| Not employed and not looking for work | 10 |
| Decline to answer | 99 |
