# Supplementary material for: Preference for Contraceptive Implant Among Women 18–44 years old
Source: Womens Health Rep (New Rochelle). 2021 Dec 15;2(1):622–32. doi: 10.1089/whr.2021.0113 (PMC8820401; doi:10.1089/whr.2021.0113)
Supplement: Supplemental data [file Suppl_TableS1.docx]

***Supplementary material***

**Supplementary Table 1. Willingness to switch contraceptive methods**

|  | **Raw Estimates** | | | | Weighted Estimates | | | | | | | | | | |
| --- | --- | --- | --- | --- | --- | --- | --- | --- | --- | --- | --- | --- | --- | --- | --- |
|  | ***Age:***  ***18 – 24***  ***N = 400*** | ***Age:***  ***25—34***  ***N = 400*** | ***Age:***  ***35 – 44***  ***N = 400*** | ***Total***  ***N =***  ***1,200*** | ***Age:***  ***18 – 24***  ***N = 8,631,355*** | | | ***Age:***  ***25—34***  ***N = 14,994,746*** | | | ***Age:***  ***35 – 44***  ***N = 14,399,008*** | | | ***Total***  ***N = 37,965,109*** | |
| **Willing to switch to another birth control form with no cost restraints** | **N (%)** | **N (%)** | **N (%)** | **N (%)** | **N (%)** | **95% CI** | **N (%)** | | **95% CI** | **N (%)** | | **95% CI** | **N (%)** | | **95% CI** |
| Yes | 347 (87%) | 348 (87%) | 328 (82%) | 1,023 (85%) | 7,466,826 (87%) | 6,669,647- 8,264,004 | 13,137,723 (88%) | | 1,1922,385-14,353,062 | 11,754,629 (82%) | | 10,573,084- 12,936,173 | 32,359,178 (85%) | | 31,269,834- 33,448,522 |
| No | 53 (13%) | 52 (13%) | 72 (18%) | 177 (15%) | 1,164,529 (13%) | 811,668- 1,517,390 | 1,857,023 (12%) | | 1,348,139- 2,365,905 | 2,584 379 (18%) | | 1,953,280- 3,215,477 | 5,605,931 (15%) | | 4,759,950- 6,451,912 |
| **Willing to switch to implant N** |  |  |  |  |  | | | | | | | | | | |
| Yes | 241 (60%) | 234 (58%) | 215 (54%) | 690 (58%) | 5,085,626 (59%) | 4,392,513- 5,778,740 | 8,818,245 (59%) | | 7,762,155- 9,874,335 | 8,039,187 (55%) | | 6,633,970- 8,641,866 | 21,541,789 (57%) | | 20,282,928- 22,800,650 |
| No | 159 (40%) | 166 (42%) | 185 (46%) | 510 (42%) | 3,545,729 (41%) | 2,959,138- 4,132,320 | 6,176,501 (41%) | | 5,268,792- 7,084,210 | 6,701,090 (45%) | | 5,734,777- 7,667,403 | 16,423,320 (43%) | | 15,196,023- 17,650,617 |
| **Willing to switch to IUD/IUS** |  |  |  |  |  | | | | | | | | | | |
| Yes | 232 (58%) | 250 (63%) | 225 (56%) | 707 (59%) | 4,985,026 (58%) | 4,296,385- 5,673,667 | 9,422,051 (63%) | | 8,336,755- 10,507,347 | 7,637,918 (55%) | | 7,081,616- 9,154,419 | 2,2525,094 (59%) | | 21,256,033- 23,794,155 |
| No | 168 (42%) | 150 (37%) | 175 (44%) | 493 (41%) | 3,646,329 (42%) | 3,053,575- 4,239,083 | 5,572,695 (37%) | | 4,707,412- 6,437,979 | 6,220,991 (45%) | | 5292869- 7,149,113 | 1,5440,015 (41%) | | 14,241,539- 16,638,491 |
| **Reasons for not considering an IUD/IUS** |  |  |  |  |  | | | | | | | | | | |
| Do not want something in uterus | 107 (64%) | 104 (69%) | 115 (66%) | 326 (66%) | 2,284,506 (63%) | 1,813,689- 2,755,322 | 3,859,152 (69%) | | 3,119,840- 4,598,466 | 4,203,101 (68%) | | 3,399,609- 5,006,592 | 10,346,759 (67%) | | 9,259,614- 11,433,904 |
| Concern over effectiveness | 37 (22%) | 25 (17%) | 29 (17%) | 91 (18%) | 827,379 (23%) | 525,912- 1,128,844 | 931,602 (17%) | | 555,172- 1,308,033 | 1,018,208 (16%) | | 624,220- 1,412,197 | 2,777,189 (18%) | | 21,256,033- 23,794,155 |
| Concern with discomfort with insertion/removal | 97 (58%) | 88 (59%) | 102 (58%) | 287 (58%) | 2,168,462 (59%) | 1,702,491 2,634,433 | 3,226,760 (58%) | | 2,554,601- 3,898,919 | 3,653,364 (59%) | | 2,914,061- 4,392,668 | 22,525,094 (78%) | | 8,031,594- 10,065,579 |
| Expected side effects | 95 (57%) | 82 (55%) | 96 (55%) | 273 (55%) | 2,189,184 (60%) | 1,725,404- 2,652,964 | 3,047,542 (55%) | | 2,386,332- 3,708,752 | 3,371,990 (54%) | | 2,675,110- 4,068,870 | 8,608,716 (56%) | | 21,256,033- 23,794,155 |
| Do not want something that lasts 3-10 years | 45 (27%) | 42 (28%) | 52 (30%) | 139 (28%) | 1,024,606 (28%) | 688,812- 1,360,400 | 1,553,628 (14%) | | 1,076,780- 2,030,475 | 1,940,637 (19%) | | 1,372,270- 2,509,005 | 4,518,871 (17%) | | 3,733,308- 5,304,433 |
| Other | 6 (4%) | 8 (5%) | 16 (9%) | 30 (6%) | 162,267 (4%) | -2,609 - 327,143 | 274,813 (5%) | | 80,424- 469,201 | 509,746 (8%) | | 259,792- 759,707 | 946,826 (6%) | | 592,261- 1,301,391 |
