# Supplementary material for: Preference for Contraceptive Implant Among Women 18–44 years old
Source: Womens Health Rep (New Rochelle). 2021 Dec 15;2(1):622–32. doi: 10.1089/whr.2021.0113 (PMC8820401; doi:10.1089/whr.2021.0113)
Supplement: Supplemental data [file Suppl_TableS2.docx]

**Supplementary Table 2. Potential Unintended Pregnancies Due to Making the Implant Unavailable**

|  | **Age 18 – 24** | **Age: 25-- 34** | **Age: 35 - 44** | Total |
| --- | --- | --- | --- | --- |
| Number of underserved women (current implant users and implant non-users willing to switch to implant) (N) | 4,763,772 | 8,562,291 | 7,236,055 | 20,562,118 |
| Number of unintended pregnancies: Current implant users on a second choice contraceptive N (%) | 47,925 (1%) | 20,323 (0%) | 45,955 (1%) | 114,203 (1%) |
| Number of unintended pregnancies: Non implant users willing to switch to an implant on a second choice contraceptive N(%) | 281,339 (6%) | 605,598 (7%) | 596,114 (8%) | 1,483,112 (7%) |
| TOTAL Unintended pregnancies due to making the implant unavailable N(%) | **329,325 (7%)** | **625,921 (7%)** | **624,069 (9%)** | **1,597,315 (8%)** |
